# Implementation of Home-Based Rehabilitation for Children With Cerebral Palsy in Douala, Cameroon: Perspectives of Caregivers and Rehabilitation Technicians

**DOI:** 10.7759/cureus.114944

**Published:** 2026-08-21

**Authors:** Gilbert Mua Akwa, Muyer Patricia Nadine Ngala, Timothy Fanfon, Hyacinte Trésor Ghassi, Dilane Landry Nsangou Muntessu, Tatuegan Womsi Ruslaine, Basil Kum Meh, Franklin Chu Buh

**Affiliations:** 1 Department of Public Health, Faculty of Medicine and Biomedical Sciences, University of Yaoundé I, Yaoundé, CMR; 2 Department of Physiotherapy, Rehabilitation Training Center of Excellence (RTCE), Baptist School of Public Health, Mutengene, CMR; 3 Department of Physiotherapy and Physical Medicine, University of Dschang, Dschang, CMR; 4 Department of Biology and Physiology of Animal Organisms, University of Dschang, Dschang, CMR; 5 Department of Physiotherapy, STEM Higher Institute of Health and Technological Sciences (STEM-HIHTS), Douala, CMR

**Keywords:** cerebral palsy, home-based rehabilitation, physiotherapy, primary caregivers, rehabilitation technicians

## Abstract

Introduction

Despite the known benefits of home-based rehabilitation (HBR) for children with cerebral palsy (CP), its implementation remains limited in most low-resource settings.

Objective

This study aimed to explore the perspectives of caregivers (CGs) and rehabilitation technicians (RTs) on HBR for children with CP. Specifically, the study assessed their experiences, challenges, and suggestions for the effective implementation of HBR for children with CP.

Methods

A phenomenological qualitative study design was used to explore the experiences, challenges, and suggestions of CGs and RTs regarding HBR and to identify strategies for its effective implementation. Participants were enrolled from the rehabilitation programme at Mboppi Baptist Hospital Douala, Cameroon. We conducted 24 interviews: 16 face-to-face and eight by mobile phone. The data were analysed using reflexive thematic analysis, which yielded six themes: Theme 1, CG perceptions of HBR; Theme 2, RT perceptions of HBR; Theme 3, challenges faced by CGs in implementing HBR for children with CP; Theme 4, challenges faced by RTs in implementing HBR; Theme 5, strategies needed by CGs for effective HBR; and Theme 6, strategies proposed by RTs to enhance HBR.

Results

The key findings highlighted challenges, such as limited resources and inadequate training, that hindered the effective implementation of HBR. RTs reported systemic and logistical challenges, such as a limited rehabilitation workforce, inadequate transportation, insufficient institutional support, and skills gaps, particularly in applying the step approach to HBR. CGs faced challenges such as limited knowledge and skills, difficulty adapting rehabilitation tasks, inconsistent support from RTs, irregular home visits by RTs, and family rejection. Continuous training, CG support, and the increased availability of skilled RTs were the main suggestions for improving HBR for children with CP.

Conclusion

The study underscores the need for structured HBR interventions, CG education, an expanded rehabilitation workforce, and policy improvements to enhance service accessibility while prioritising a family-centred approach. These findings will inform healthcare planning and optimise rehabilitation for children with CP in Cameroon and similar settings.

## Introduction

Cerebral palsy (CP) comprises a group of permanent disorders of movement and posture caused by non-progressive disturbances in the developing foetal or infant brain [1,2,3]. This condition is frequently accompanied by sensory, cognitive, communicative, and behavioural impairments, as well as epilepsy and secondary musculoskeletal problems [3-6]. Children with CP often face significant challenges in social participation, autonomy, and self-efficacy, which hinder their ability to perform daily activities [3,7]. Globally, neurodevelopmental disorders, including CP, are estimated to affect up to 9.3% of individuals under 18 years of age [4]. CP is the largest diagnostic group treated in paediatric rehabilitation. Despite increased survival rates among infants with low birth weight, physiotherapy services and rehabilitation centres remain limited in most low- and middle-income settings [8]. According to global data, the birth prevalence of pre- and perinatal CP was as high as 3.4 per 1,000 live births (95% CI, 3.0-3.9) in low- and middle-income countries (LMICs), compared with 1.5 per 1,000 live births (95% CI, 1.4-1.6) in high-income countries (HICs) [9]. In Africa, reported prevalence rates are notably high, ranging from 2 to 10 per 1,000 children [10]. A Ugandan study reported a prevalence of 2.9 per 1,000 children, while a South African study reported a higher prevalence of 10 per 1,000 children [9]. Although epidemiological data are limited in Cameroon, the study by Mangamba DK et al. [11] reported a CP prevalence of 4.86% among 4,064 medical records at the paediatric department of the Douala Gyneco-Obstetric and Paediatric Hospital. At the same hospital, Enyama D et al. [12] found a CP prevalence of 31.3% among paediatric neurological disorders at the paediatric neurology clinic, making it the second most prevalent disorder after epilepsy. However, these rates were reported by a single referral hospital and may not reflect the actual situation in the country. Thus, comprehensive epidemiological data on the burden of CP remain limited, even though these cases are frequently encountered in physiotherapy services and rehabilitation centres throughout the country.

Effective rehabilitation is paramount for integrating children with CP into their home, school, and social environments. They require technological and support services, facilitated by multidisciplinary healthcare teams, to achieve social integration [13,14]. Children with CP require highly specialised care involving a collaborative group of healthcare providers and CGs, including paediatricians, rehabilitation medicine physicians, nurses, general practitioners, orthopaedic surgeons, nutritionists, speech and language therapists, occupational therapists, multiskilled rehabilitation technicians (RTs), community-based rehabilitation (CBR) workers, and physiotherapists [15]. However, this comprehensive care may not be affordable in many LMICs, including Cameroon; therefore, rehabilitation models that may benefit LMICs have been developed.

To date, various models of rehabilitation service delivery have been proposed to provide affordable and appropriate support to children with disabilities. These can be broadly categorised into institution-based rehabilitation (IB), CBR, and outreach-based rehabilitation (OR) approaches. Home-based rehabilitation (HBR) is one prominent rehabilitation strategy adopted worldwide for various clinical conditions, including CP, and is supported by substantial evidence [16]. However, it remains understudied in LMICs, particularly in sub-Saharan African settings, where the burden of CP is substantial. HBR can account for 50-80% of the total therapy received by children with CP and has been shown to improve not only children’s progress but also parental satisfaction [17]. Moreover, it is valuable to families because it saves time and resources, involves parents in treatment decisions, and enables them to choose the most suitable options for their children [17]. HBR generally includes activities performed by CGs and/or therapists to promote active movement, improve functional range of motion, strengthen weakened muscles, and support motor development, thereby preventing or reducing complications such as joint contractures, pain caused by spasticity, and reduced active movement associated with CP [18,19]. Developing functional skills in children with CP requires repeated practice in various environments, including the home, which cannot be achieved solely through traditional face-to-face therapy in rehabilitation centres [8]. Despite growing evidence supporting the effectiveness of community-based services and HBR in managing CP, implementation remains limited in most LMICs, including Cameroon. Evidence on HBR is limited or nonexistent in Cameroon and Central Africa more broadly. Exploring the perspectives of CGs and RTs on implementing HBR would provide context-specific data that could inform strategies to improve the care and clinical outcomes of children with CP. Thus, this qualitative study aimed to assess the perspectives of CGs and RTs on HBR for children with CP in Cameroon.

## Materials and methods

Study site

This study was conducted at Mboppi Baptist Hospital Douala (MBHD), which provided HBR services for children with CP in Douala, Cameroon. The hospital is situated in a highly cosmopolitan city characterised by diverse socioeconomic backgrounds and cultural beliefs, which significantly influence the management of CP. MBHD was selected because it was the only hospital in Douala and one of the few hospitals in Cameroon that extended rehabilitation services into communities through community-based rehabilitation programmes.

Research design

This study adopted a phenomenological qualitative research approach to explore the lived experiences of CGs and RTs regarding HBR for children with CP. The study was based on in-depth interviews with CGs and RTs. This approach allowed for an in-depth understanding and identification of the challenges associated with the effective implementation of HBR for children with CP [8].

Study period 

The study was conducted from May to July 2025. Data collection took place from June 1 to June 30, 2025. From March to May 2025, the research proposal, including the interview guides, was developed.

Study population

The study population comprised parents, guardians, or other family members who were the primary CGs of children with CP, resided in Douala I, II, or III, and were registered in the rehabilitation outreach programme at MBHD.

The study also included RTs working at MBHD, namely physiotherapists, physiotherapy assistants, community-based rehabilitation workers, and multiskilled RTs.

Inclusion Criteria

The inclusion criteria for CGs were being the primary CG of a child diagnosed with CP; being registered with MBHD and residing in a community visited by healthcare providers from the Cameroon Baptist Convention Health Services (CBCHS) for community-based rehabilitation, specifically Douala I, II, or III; being able to communicate in French, English, or Pidgin English; and signing a consent form indicating their willingness to participate in the study. Douala I, II, and III are three of the six subdivisions that constitute Wouri Division. They are closest to MBHD and are readily accessible to the RTs involved in community-based rehabilitation.

The inclusion criteria for RTs were employment at MBHD, at least one year of work experience as a physiotherapist, physiotherapy assistant, multiskilled RT, or community-based rehabilitation worker, and provision of consent to participate in the research.

Exclusion Criteria

The exclusion criteria were RTs who were unavailable at the scheduled interview times and did not attend rescheduled interviews during the study period, CGs who were not in a good general condition at the time of the interview, and individuals who were not the primary CGs of children with CP.

Sampling Size Determination

The planned sample size was 20-30 participants. This range was considered adequate to allow for data saturation based on previous similar studies [20,21]. The final sample comprised 24 participants: 20 CGs of children with CP and four RTs.

Sampling Technique and Participant Recruitment

A consecutive sampling technique was used to recruit participants who met the inclusion criteria, ensuring the consideration of individuals directly involved in caring for children with cerebral palsy, either as primary CGs or RTs. Consecutive sampling was chosen because all eligible participants were approached during the study period and enrolled after signing the consent form. The researcher contacted eligible CGs and RTs, and a mutually convenient interview time was agreed upon and scheduled. Recruitment of CGs ended after the target of 20 participants was reached, and all four available RTs were recruited.

Coding

Participant identification codes were assigned to each participant. CGs were assigned codes P1-P20, while RTs were assigned codes P21-P24. The audio files were labelled using these codes, all transcripts were documented, and responses were organised according to thematic areas for each participant. All research team members contributed directly or indirectly to selecting the analytical codes reported in the manuscript. The interviewer and supervisor, together with one member of the research team, initially selected the codes reported under the various themes. These were presented to the remaining team members, who were given an opportunity to suggest adjustments. A few additional codes were added based on their recommendations.

Data collection

Semi-structured interview guides were used to explore CGs’ views, experiences, challenges, and support needs (Appendix 1), as well as RTs’ roles, strategies, challenges, and best practices (Appendix 2).

The interview guides were developed by defining the core research questions and translating them into specific open-ended questions. The questions were designed around the key aspects explored in the study: experiences with HBR for children with CP, challenges, and suggestions for its effective implementation. Members of the research team met and drafted the interview guides, which were subsequently reviewed and corrected before the final versions were produced. Care was taken to ensure that the interview guides addressed the core research questions. The interview guides were pretested with two randomly selected primary CGs and one RT at Baptist Hospital Mutengene. This was done to ensure that the tools adequately explored the experiences, challenges, and suggestions of CGs and RTs regarding HBR in the care of children with CP.

A short structured questionnaire was used to collect demographic information from both CGs and RTs. In-depth interviews were conducted face-to-face or by telephone. The study objectives were discussed with the CGs and RTs, and they were given an opportunity to ask questions and seek clarification to improve their understanding of the study. Informed consent was obtained from all participants. Participants were informed that their interviews would be audio-recorded from the outset. The interviews were recorded using an Android mobile phone, and the recordings were transferred to a laptop and stored in a password-protected folder to maintain confidentiality. The interviews lasted 35-45 minutes. Care was taken to ensure that the interviewer did not have a clinical relationship with the CGs, thereby minimising potential bias. The interviewer ensured that the participants understood the questions being asked. Terms such as rehabilitation and HBR were also explained to the CGs to ensure the accuracy of their responses.

Data management

All 24 audio recordings, comprising interviews with 20 CGs and four RTs, were manually transcribed and stored in two folders on a computer. The folders were labelled “CG” for caregivers and “RT” for rehabilitation technicians to avoid confusion. The data were stored in password-protected cloud storage and on a password-protected laptop to prevent unauthorised access by individuals outside the research team. Participants were assigned identification codes P1-P20 for CGs and P21-P24 for RTs.

Data analysis

The interviews were audio-recorded in either English (n = 20) or French (n = 4), depending on each participant’s preferred language. All interviews were transcribed verbatim, and the French transcripts were translated into English. The transcripts were read, and the audio recordings were replayed several times to ensure transcription accuracy. The French transcripts were translated into English using Google Translate and reviewed by two members of the team, the interviewer and the supervisor, who were fluent in both French and English, to ensure accuracy. A codebook was created for all transcripts, and the transcripts were analysed using thematic analysis. The analysis began with familiarisation through listening to the audio recordings and reading and rereading the transcripts. Reflexive thematic analysis was used to analyse the data and generate themes. The analysis focused on developing themes related to the perspectives of CGs and RTs on the effective implementation of HBR for children with CP, the challenges they faced, and strategies for improving HBR at MBHD. The six themes were as follows: Theme 1, CG perceptions of HBR; Theme 2, RT perceptions of HBR; Theme 3, challenges faced by CGs in implementing HBR for children with CP; Theme 4, challenges faced by RTs in implementing HBR; Theme 5, effective strategies needed by CGs for HBR; and Theme 6, strategies proposed by RTs to enhance HBR.

Reflexivity

Team reflexivity was undertaken in accordance with the seven orienting questions outlined by Barry CA et al. [22]. All authors of this paper are either physiotherapists or physicians. The interviewer (MPNN) is a certified physiotherapist working under the CBCHS, with a specific interest in qualitative research. The interviewer is from an English-speaking region of Cameroon but grew up and received most of her education in the French-speaking part of the country, making her fluent in both languages. She reads, speaks, and writes both official languages of Cameroon: English and French. The supervisor (FCB) is a lecturer and an experienced physiotherapist with expertise in neurological rehabilitation. The remaining physiotherapists hold at least an MSc degree, while GMA is a physician. The authors adopted a phenomenological approach, recognising that knowledge acquisition is influenced by environmental and social interactions [23].

Ethical considerations

Ethical approval for the study was obtained from the Institutional Review Board (IRB) of the CBCHS, reference no. IRB2025-58. Administrative authorisation was obtained from MBHD, reference no. CBC/MBHD/ADM-L/25/143. The study was conducted in accordance with the principles outlined in the Declaration of Helsinki for medical research involving human participants. CGs and RTs provided written informed consent before participating in the study. The data were kept confidential, and participants’ identities were protected. Participants had the right to withdraw from the study at any time and received detailed information about the study’s purpose, methods, and their rights.

Reporting

The study was reported in accordance with the Consolidated Criteria for Reporting Qualitative Research (COREQ) guidelines [24]. COREQ is a 32-item checklist for reporting qualitative studies involving interviews and focus groups.

## Results

Participant characteristics

**Figure 1:**
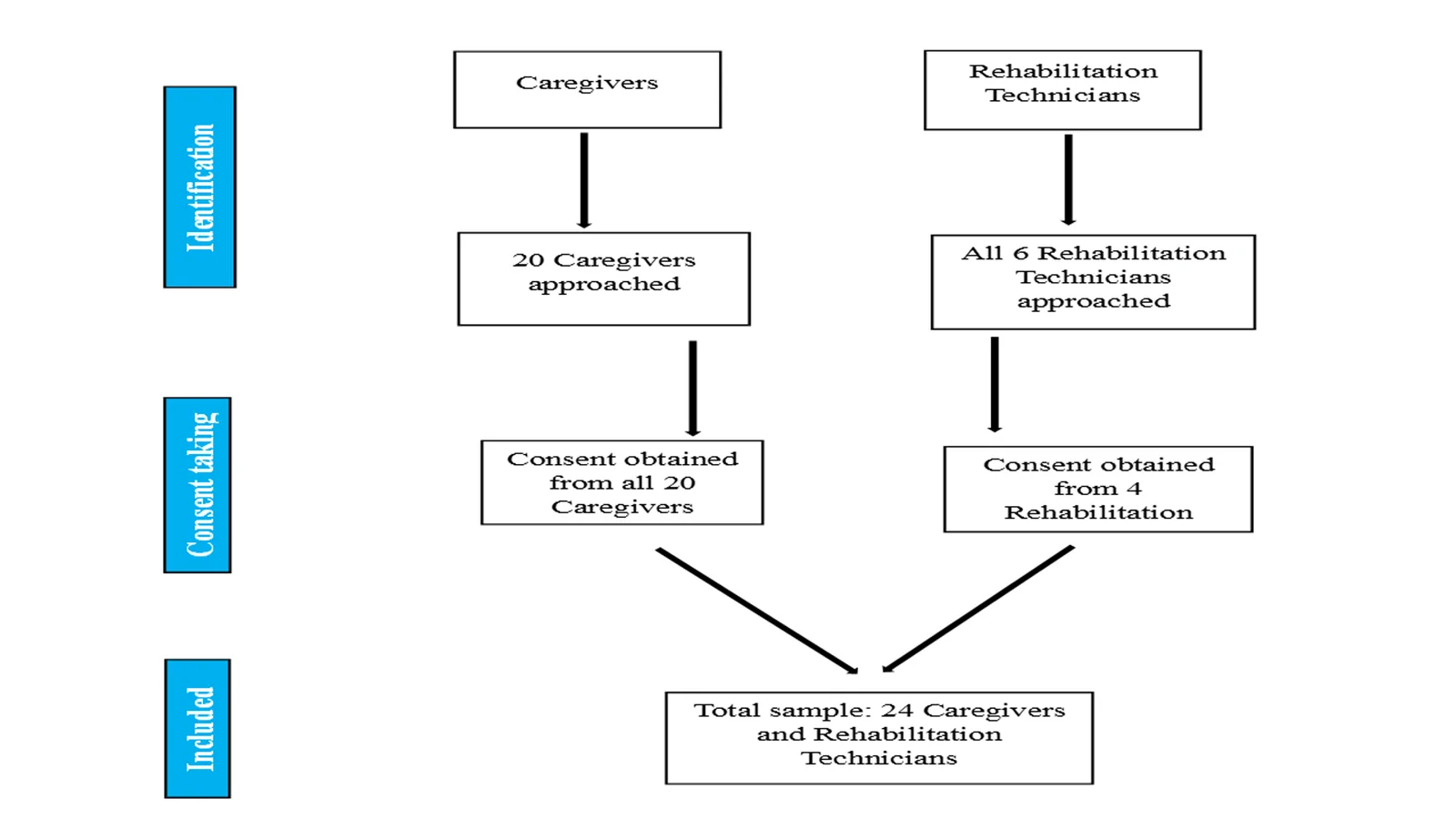
Participants included in the study.

A total of 26 participants were approached to take part in the study: 20 CGs and all six RTs involved in community-based rehabilitation programmes. All 20 CGs and four RTs agreed to participate. Therefore, 24 participants were included in the analysis (Figure 1). Of these, 16 were interviewed face-to-face and eight were interviewed by telephone. Twenty participants were interviewed in English, while four were interviewed in French.

CG and CP Children Characteristics

Most children with CP were male (11; 55%). The age groups of 0-10 years and 11-19 years were equally represented, with 10 children (50%) in each group. The most represented age group among the CGs was 36-45 years (50%). Regarding educational level, most CGs (12; 60%) had attended secondary school. Regarding occupation, most CGs (12; 60%) were traders. Most CGs (17; 85%) were the parents of the children with CP. All CGs who participated in the study were female (20; 100%), as shown in Table 1.

**Table 1:** Sociodemographic characteristics of children with CP and their caregivers (20 CGs).

Variable	Category	Frequency (n)	Percentage (%)
Children’s sex	Male	11	55
	Female	9	45
Children’s age group (years)	0-10	10	50
	11-19	10	50
Caregivers’ age group (years)	<25	1	5
	26-35	6	30
	36-45	10	50
	>45	3	15
Caregivers’ level of education	Primary	3	15
	Secondary	12	60
	Tertiary	5	25
Profession	Business	12	60
	Homemaker	3	15
	Teacher	2	10
	Nurse	2	10
	Retired	1	5
Relationship to child	Parent	17	85
	Aunt	2	10
	Grandmother	1	5

RT Characteristics

Most RTs were female (3; 75%), and the majority were diploma holders (3; 75%); only one (25%) held a bachelor’s degree. Regarding specialty, one RT (25%) was a community-based rehabilitation worker, one (25%) was a physiotherapy assistant, one (25%) was a physiotherapist, and one (25%) was a multi-skilled RT (Table 2).

**Table 2:** Demographic characteristics of rehabilitation technicians (4 RTs). RT: Rehabilitation technician.

Variable	Category	Frequency (n)	Proportion (%)
Sex	Male	1	25
	Female	3	75
Qualification	Diploma	3	75
	Bachelor’s degree	1	25
Specialty	Community-based rehabilitation worker	1	25
	Physiotherapy assistant	1	25
	Physiotherapist	1	25
	Multi-skilled rehabilitation technician	1	25

Themes

Thematic analysis was used to identify patterns and insights, which are presented under six major themes.

Theme 1: CG Perceptions and Experiences of HBR

CGs expressed varied perceptions of HBR for children with CP. Many viewed it as a vital support system that promotes the child’s independence and engagement within the family. They also believed that rehabilitation at home enhances the child’s comfort and emotional well-being, thereby encouraging more active participation in rehabilitation. Despite the challenges, the majority of CGs reported positive experiences with HBR and appreciated its convenience, accessibility, and the empowerment it provided in managing their child’s condition. However, dissatisfaction arose when follow-up was irregular and when CGs felt inadequately equipped with the skills and materials needed for home-based exercises. Participant narratives emphasised the dual nature of these experiences, with HBR being both a lifeline and a source of stress, depending on the level of support received.

Participant P13 reported, “HBR is very important for my family and I because my children also learn when the therapist comes and they also practice the things they learn on their sister. I feel empowered and encouraged each time they pass around for rehabilitation, though the outcome is slow, but I am happy that there are always little improvements especially now that the seizures have been controlled. Also, HBR is better than hospital-based rehabilitation because the environment is user friendly, accessible, convenient and comfortable.”

Similarly, Participant P1 stated “HBR is very important because when the rehabilitation technicians visits us, all the members in my family watch and learn the techniques from the RT and later practice on the child hence reducing the burden of one person carrying the child to the hospital which is stressful, costly and time consuming.”

Experiences of HBR were not positive for all participants, as some reported feeling frustrated by their lack of skills in performing exercises with their children and by the severity of their children’s CP. For example, Participant P12 stated:

“I feel lost, frustrated, and hopeless when performing exercise on my child, because I am not skilled enough to rehabilitate my child. My child has severe CP and are 12 years old. She needs assistance in all aspects. I become tired, stressed up, and even experience burnout since I have to focus all my attention on this child. So, I need a guide and intensive follow-up from the RTs to help my child.”

Theme 2: RT Experiences and Perceptions of HBR

RTs generally echoed the view that HBR interventions foster greater family involvement in the rehabilitation process, which is crucial for promoting the child’s development and community integration.

Participant P24 noted that “HBR is very vital for both the child and family. When the rehabilitation plan is family-centered and unique to that child, it helps CG to also adhere to the rehabilitation program. It also helps the family to work together to help the child and reduce the stress and burden of one person always carrying the child to the hospital for the rehabilitation process. However, HBR needs to be strengthened by regular home visits by the RTs, creating a multidisciplinary team for HBR, organizing workshops for both CG and RTs, and recruiting more RT staff.”

Participant P23 reported “The STEP program has to come to elevate HBR; it makes work easy as we empower many parents of children with disability, including CP. I can track the rehabilitation outcome using the STEP approach.”

The Support Tools Enabling Parents (STEP) programme ensures that the care of a child with CP is broken down into small steps. To walk, a child must first learn to sit and then crawl before standing and walking. Under this approach, children with CP must also learn to eat and drink properly, use the toilet, communicate, play, and interact with other children [25].

Theme 3: Challenges Faced by CGs in Implementing HBR for Children with CP

CGs described various challenges encountered when implementing HBR, including limited CG knowledge and skills, difficulty adapting rehabilitation tasks, inconsistent support from RTs, irregular home visits by RTs, inadequate training in exercise techniques, insufficient assistive devices, family rejection, uncontrolled seizures, and cultural beliefs and stigma. Limited space at home for rehabilitation exercises and limited access to specialised personnel, including RTs, were also highlighted as challenges.

Participant P13 explained “In my opinion, I need more skills and training, specifically in rehabilitating my child, even the therapist, some of them still practice the old way of rehabilitation, which is not effective, when I compare the rehabilitation activities I watch online and the ones they practice on my child. Gaining these skills will improve the outcome of rehabilitation.”

Participant P5 lamented that inconsistent follow-up of her child placed her in a stressful situation and that she sometimes felt discouraged that the child would not achieve autonomy. Her original statement was translated from French into English as follows: “The lack of consistency in my child’s follow-up leaves me feeling helpless, frustrated, and sometimes discouraged; since my child can neither walk nor do anything for herself, this leads to less progress and prevents her from achieving independence.”

Participant P3 reported, “Since they identify my child, nobody has come to do rehabilitation on the child. Most often, I do stretches and massages on my child, and it has not been easy because I don’t know if I am doing the right exercises for my child.”

Participant P12 stated that the rehabilitation professional visited her child only once or twice per month, spent little time performing exercises, and sometimes visited only to administer medication from the hospital. Her original statement was translated from French into English as follows “The field worker stops by once or twice a month for follow-ups, and sometimes doesn’t come at all. They devote very little time to exercises with my child, sometimes coming only to administer medication. This makes things extremely difficult for me.” CGs also faced rejection of children with CP, and sometimes rejection of the CGs themselves, by other family members. For example, other family members might not feed or clean the child when the CG was not at home. CGs also highlighted their limited skills in implementing HBR. The severity of the child’s condition and uncontrolled seizures were other challenges encountered.

Participant P7 said, “My family thinks my child is a curse and the child can contaminate their children, even my landlord sent me out of the house where I was renting, and I had to look for a house where it will only be my children and me.”

Limited space at home for rehabilitation exercises and limited access to specialised personnel, such as speech and language therapists and occupational therapists, were also highlighted as challenges. These findings suggest the need for and importance of multidisciplinary care for these children.

Participant P7 said, “My child struggles to speak, and sometimes she uses gestures or actions to communicate. She likes to go to school, and she struggles to write with the affected hand. After reading on the internet, I realized that other specialists such as speech and occupational therapists could help my child’s situation. So, I think if there were other specialists such as a speech therapist and occupational therapist, I believe my child could have been talking and using the affected hand by now.”

Theme 4: Challenges Faced by RTs in Implementing HBR

RTs also described challenges in implementing the programme, some of which mirrored those reported by CGs. Limited staffing and a lack of institutional support were common challenges faced by RTs when implementing HBR for children with CP. One participant said, “Many rehabilitation technicians still have difficulties using the STEP approach, which makes work difficult and challenging” (P23).”

RTs reported that limited staffing made it difficult to provide HBR effectively. They also highlighted that some RTs lacked the skills required to care for children with CP at home. Participant P23 said, “Averagely in a day we receive about 30 outpatients, and patient to staff ratio is approximately 1:6 on a daily basis, this makes it difficult for us to even go out to the community to rehabilitate these children, this hinders the effectiveness of HBR of children with CP.”

Participant P22 explained, “My first experience in the community was frustrating and challenging because I realized that I didn’t have the skills to do HBR on children with CP.”

RTs also reported poor adherence by children and CGs to treatment and the absence of a multidisciplinary team in their departments, making it difficult to implement HBR effectively. Participant P21 reported, “If a multidisciplinary team is created for HBR, it will become easier and reduce the stress of the CG because sometimes the transportation means for the caregiver to visit all the departments I refer them to is quite expensive since each department or a specialist may have a particular day to consult and each specialty has its consultation fee. Additionally, some of these children do not walk and are fully dependent, which strains the caregiver a lot by carrying the child from one specialist to the other and on different days, this always led to poor adherence to the rehabilitation plan.”

Furthermore, RTs highlighted difficulties in completing treatment because some CGs sought medical care for their children at a later age, making favourable treatment outcomes more difficult to achieve. Poor adherence to treatment by CGs and children also hindered the progress of HBR, as some CGs did not follow up with their children at home according to the RTs’ instructions.

Participant P21 explained, “Some parents do not follow instructions, as they will say, ‘I was told that is a spiritual problem, so I had to take my child to church for prayers, and I also forgot to do the exercises. I no longer give the drugs to my child because it makes the child too weak.’ This makes rehabilitation more difficult, and the goal for that child may not be achieved. In cases like that, I have to do counselling again to make sure that the parent understands the child’s problem.”

Theme 5: Effective Strategies Needed by CGs for HBR

Participants generally perceived the existing HBR strategies as beneficial but expressed a desire for more tailored approaches. CGs noted that although they appreciated the structure of HBR, they would benefit from individualised plans tailored to each child’s unique needs. Based on their experiences, CGs suggested several strategies for effectively implementing HBR. They suggested that receiving additional training and developing skills in caring for children with CP at home would allow them to implement HBR more effectively. Participant P15 said, “My child was identified when he was 4 years and could not walk, but when they used the step approach by assessing the environment and they worked closely with us, after 4 months my child was able to walk using parallel bars.”

CGs also highlighted the need for frequent visits by RTs to evaluate the activities performed with the children and provide support to optimise care and outcomes. Assistive equipment, such as parallel bars, was also suggested as an effective strategy. Participants also suggested organising additional training sessions and workshops for CGs. CGs emphasised that regular home visits by RTs were a stepping stone towards the effective implementation of HBR in CP management.

Participant P13 stated, “Consistency, duration, and frequency of HBR are prerequisites for a better rehabilitation outcome. Follow-up should be done at least twice a week rather than once a month.”

Participant P6 said, “Workshops should be organised to train us, the CGs, on recent rehabilitation approaches; this will also improve the efficacy of HBR.”

Participant P18 reported, “The rehabilitation technicians should engage in regular follow-up, duration, and frequency of rehabilitation matters. More so, it is only one person who comes, and I think many more rehabilitation technicians should be recruited, such as speech therapy, occupational therapy, and many other specialties. This will make HBR more effective.”

Theme 6: Strategies Proposed by RTs to Enhance HBR

RTs proposed various strategies for improving HBR, including the provision of ambulatory aids, as also suggested by the CGs. They also emphasised the need to provide CGs with continuous emotional support, as CGs are often overwhelmed by caring for these children alongside their other responsibilities. With little or no family support, they may be at risk of experiencing depression.

Participant P21 noted that “Workshops should be organized on how to rehabilitate children with CP, and a multidisciplinary team should be created for HBR. This will reduce the cost of frequent visits of CG and patients going to the hospital for different appointments to meet the various specialists.”

Referring to the same child described by caregiver P15 in Theme 5, Participant P24 stated, “There is a child that we identified when he was 4 years and could not walk, but when we used the step approach by assessing their environment and the parent worked closely with us, after 4 months the child was able to walk using parallel bars.”

## Discussion

This study aimed to assess CGs’ and RTs’ perspectives on the effective implementation of HBR for children with CP in Douala, Cameroon. In this study, both CGs and RTs valued HBR but identified gaps that limited its effective implementation. The study enrolled CGs and RTs, and all the CGs were female. This predominance of female CGs was consistent with our observations and experiences in clinical practice. Female CGs, including mothers, aunts, and grandmothers, are usually those who bring children with CP to hospitals and spend most of their time caring for them. Some are isolated by their spouses or other family members. Male family members, on the other hand, are often more involved in providing financial support but generally less involved in the direct care of these children, as they may need to work and provide for the family’s financial needs. The largest age group among the CGs was 36-45 years (50%). Most RTs were female, and most were diploma (DIP) holders. The RTs included physiotherapists, physiotherapy assistants, CBR specialists, and multi-skilled RTs.

CGs’ and RTs’ experiences and perceptions of HBR

Regarding the experiences of CGs and RTs with HBR, the majority of CGs reported positive experiences, appreciating its convenience, accessibility, and the empowerment it provided in managing their child’s condition. However, dissatisfaction arose when follow-up was irregular and when CGs felt inadequately equipped. Participants’ narratives emphasised the dual nature of their experiences, with HBR being both a lifeline and a source of stress, depending on the level of support received. These findings align with those of Demeke ZD et al. [19], who concluded that CG empowerment is a decisive factor in the success of HBR programmes in low-resource settings, and Niyonsenga J et al. [16], who highlighted the role of familiar settings in enhancing therapeutic experiences for children with CP. The findings from our study are also consistent with those of Lonn H et al. [26] in Cameroon, where a lack of social support and limited access to effective rehabilitation services were identified as major challenges faced by CGs in caring for children with CP. However, our findings are more specific to the challenges associated with HBR than to the overall care of children with CP. Irregular follow-up of children with CP by RTs highlights the significant shortage of these professionals, especially in low-income settings where the burden of CP is high [27]. This corroborates reports by the WHO that there are fewer than 10 rehabilitation professionals per million population in many low- and middle-income settings [28]. Sub-Saharan African countries, including Cameroon, face substantial shortages of rehabilitation professionals. It is therefore crucial for this region to scale up the training of these professionals to meet the need for regular HBR for children with CP, which could improve outcomes and reduce the already substantial burden of the condition.

RTs generally perceived HBR as very helpful for CGs and children with CP, as the children felt comfortable in the home environment. However, their experiences with HBR were not without challenges. They advocated for more regular visits to children and strongly believed that multidisciplinary teams needed to be established because children with CP may have other problems requiring professionals such as speech and language therapists and occupational therapists. These findings are consistent with those of Demeke ZD et al. [19] in Ethiopia, where physiotherapists did not adequately monitor adherence to home-based therapy for children with CP because of irregular home visits. This may be explained by the above-mentioned shortage of RTs, as they may be more frequently deployed in hospital centres than allocated to home-based care. Furthermore, multidisciplinary rehabilitation is considered a central approach to the rehabilitation of children with CP [29,30]. According to the RTs’ responses, such teams do not exist locally, constituting a major barrier to the effective implementation of HBR for children with CP. The situation in Cameroon regarding multidisciplinary rehabilitation teams for major neurological conditions such as CP is similar to that in many other Sub-Saharan African countries and LMICs. This underscores the need for urgent, context-specific measures to support the effective implementation of HBR for children with CP.

Challenges faced by CGs and RTs in HBR

CGs reported multiple challenges hindering effective HBR. The most prevalent issue was irregular follow-up by RTs, followed by a lack of skills among CGs, family rejection, limited space at home, and the severity of the child’s condition. These findings are similar to those reported by Mwinbam MM et al. [31], who found that CGs faced arduous tasks when caring for children with CP because of socioeconomic barriers and cultural challenges. In a systematic review by Beckers LE et al. [7], there was substantial variability in treatment approaches and in the frequency and duration of interventions for CP. This indicates that although HBR may effectively improve rehabilitation outcomes in CP, there is a crucial need to address strategies for its effective implementation. Furthermore, this rehabilitation approach appears to be parent-friendly, as it was not associated with increased stress among parents. The study by Palomo-Carrión R et al. [32] indicated that early home-based interventions improved bilateral manual performance in children with CP. Despite growing evidence regarding the effectiveness of HBR for children with CP, its implementation remains slow. This problem may be worse in LMICs, where many settings have fewer than 10 rehabilitation professionals per million population [33]. Addressing the shortage of rehabilitation professionals would be a stepping stone towards providing regular HBR for children with CP.

Another key challenge faced by CGs in implementing HBR was limited family support resulting from the rejection of children with CP and sometimes the CGs themselves. This implies that rehabilitation sessions may not take place when the children’s primary CGs are ill or absent, which can substantially affect treatment sessions and rehabilitation outcomes. CGs also highlighted that they had limited skills in implementing HBR, particularly in determining what they could do to assist the children given the irregular visits by RTs. The severity of the child’s condition, such as frequent uncontrolled seizures, also constituted a barrier to HBR. When faced with this situation, some CGs preferred hospital-based rehabilitation because they believed that complications such as seizures could be better managed if they occurred during therapy. These findings may help guide the triage of children with CP who could receive HBR and those for whom more hospital-based sessions may be recommended. These findings align with those of Chiluba and Mayo [4], who reported that CGs in low-resource settings faced emotional burdens that compromised the quality of care.

Family stigma, as highlighted in a participant’s narrative, exacerbates CG isolation and emotional distress. In this setting, education is often provided only to the CGs who bring the children to the hospital, which may limit its expected impact. It is crucial to develop more inclusive family education strategies, especially for family members living in the same household as the affected child and those who visit regularly. Such education may improve their knowledge and perceptions of CP and encourage them to adopt positive behaviours that support HBR implementation and treatment outcomes.

Regarding the challenges faced by RTs, systemic and logistical barriers were the most prevalent. These included limited means of transportation, a lack of institutional authorisation, insufficient staffing, and skills gaps, particularly in applying the STEP approach to HBR. This is consistent with the findings of Oguntade HA et al. [15], who identified inadequate institutional support and insufficient specialised training as critical constraints for RTs in LMICs. As mentioned above, the rehabilitation workforce in LMICs is substantially limited, and the shortage may be particularly severe in Central Africa. Consequently, hospitals and rehabilitation centres may restrict hospital-based rehabilitation professionals from conducting home visits for therapy. This problem is aggravated by the limited availability of CBR services in many LMICs and Central African countries, including Cameroon. It is therefore important to establish and strengthen CBR services, which could facilitate the implementation of HBR for children with CP.

RTs also reported lacking the skills required to care for children with CP in a home environment. This is consistent with the findings of Albishi AM et al. [34], who reported that the home working environment constituted a barrier to rehabilitation professionals effectively implementing home-based therapy. CP is common in low-resource settings [35], where homes may have limited space for rehabilitation. This may be challenging for RTs who are accustomed to the comparatively better working conditions available in hospitals. Some may therefore experience difficulties adapting and designing home-based exercises that are feasible within the child’s environment. This finding may inform further training for RTs involved in HBR on adapting care to the different environments commonly found in low-resource settings.

Lastly, RTs highlighted poor adherence to treatment among CGs and the absence of multidisciplinary teams in their departments, making it difficult to implement HBR effectively. Further studies could investigate the factors associated with poor adherence among parents or CGs to HBR. Multidisciplinary teams are highly recommended for the care of complex neurological conditions such as CP. The RTs involved in this study focused mainly on physical rehabilitation, while some children had other problems, including speech, swallowing, and hearing difficulties; however, no specialised professionals were available to address these problems. To date, much of Africa continues to face substantial challenges in establishing multidisciplinary teams because of limited resources. Buh FC et al. [36] recommended transdisciplinary rehabilitation teams, which may be better suited to low- and middle-income settings. It is crucial to involve specialised professionals according to the problems experienced by children with CP. While physical rehabilitation professionals, such as physiotherapists and occupational therapists, may be more involved in caring for children with CP who have motor problems, speech and language therapists are indispensable for addressing speech and swallowing difficulties.

Overall, two major challenges can be identified from those reported by CGs and RTs: a lack of resources and a lack of societal support for children with CP. In the interim, parent education, workshops, and emotional support should be considered key options for improving CP rehabilitation outcomes. The active involvement of religious organisations could also help educate communities about social attitudes and values, potentially reduce the stigmatisation of families with children with CP, and improve the mental health of CGs.

Strategies to improve HBR by CGs and RTs

CGs highlighted the following key strategies to enhance HBR: additional skills training, frequent home visits by RTs, government support, and the provision of assistive devices such as parallel bars and ambulatory aids. These findings underscore the importance of empowering CGs through structured training programmes, a strategy supported by Beckers et al. [7] for sustainable rehabilitation outcomes. RTs echoed the needs of CGs but further emphasised broader systemic interventions. The strategies proposed included continuous training workshops, emotional support for CGs, increased community awareness campaigns on CP, regular home visits by RTs, and expansion of the rehabilitation workforce through the training and recruitment of additional RTs. These findings suggest a critical need for policy reforms that facilitate multidisciplinary collaboration and community engagement to improve CP rehabilitation outcomes. The similarity between the suggestions of RTs and CGs reflects their shared aspirations for the effective implementation of HBR and improved outcomes for children with CP. HBR could benefit most CGs by reducing transportation costs to health facilities, providing a less stressful and anxiety-provoking environment, and reducing waiting times compared with hospital-based care [37]. Addressing the challenges highlighted by RTs and CGs and considering their suggestions could increase adherence to HBR among CGs and children with CP and thereby improve rehabilitation outcomes.

Limitations and strengths

The study was limited in scope, as participants were recruited from Mboppi Baptist Hospital Douala in Cameroon’s economic capital. This may limit the generalisability of the findings across Cameroon. Another major limitation was that the clinical characteristics of the CGs’ children were not considered, although such characteristics could influence the experiences of the CGs. Most participants also resided in urban areas, which may affect the generalisability of the findings to rural areas, where social realities may differ. Not all interviews were conducted face-to-face; some were conducted by telephone. This variation in the interview method may have affected how participants responded to the questions. The small number of RTs enrolled in the study may also have resulted in some relevant experiences being missed. However, this may reflect the limited availability of CBR services, as Mboppi Baptist Hospital is one of the few, if not the only, hospital centres with CBR activities in Douala. Although only four RTs participated, all six RTs involved in this programme were approached. This study focused mainly on CGs and RTs. Although their perspectives are crucial, including insights from other relevant stakeholders, such as physicians and policymakers, could provide a more comprehensive understanding of the challenges and help foster the effective implementation of HBR.

Nevertheless, this study provides a snapshot of the experiences of CGs of children with CP and rehabilitation professionals involved in HBR and, to our knowledge, is the first such study in the Central African region. The findings of this study may also provide a basis for policy improvements in CBR, which remains limited in many LMICs, including Cameroon.

Novel clinical implications for CGs and RTs in HBR of children with CP

Education and effective communication with the families of children with CP must be emphasised to help combat stress and anxiety and build the caregiver resilience needed to support these children throughout long-term rehabilitative care. Community-specific education about CP is also crucial and should be implemented to address cultural and social beliefs that hinder the effective implementation of HBR. Cultural beliefs can constitute a major source of emotional stress among CGs. For example, P7 expressed frustration after being rejected by her family and asked by her landlord to leave her home. With effective community education, such practices could be substantially reduced. There is also a crucial need to increase the skilled rehabilitation workforce in low-income settings. The goals of the WHO Rehabilitation 2030 initiative cannot be achieved without addressing the extremely limited rehabilitation workforce in low- and middle-income settings, where the burden of disability is high. Increasing the number of rehabilitation workers may reduce institutional barriers to allocating rehabilitation workers to community health services. It is also crucial to establish multidisciplinary or transdisciplinary HBR teams comprising other professionals, such as speech and language therapists and occupational therapists, who could help address the speech and writing difficulties experienced by affected children. Despite the limited number of rehabilitation professionals in low-resource institutions, hospitals and rehabilitation centres need to support the scalable implementation of HBR for children with CP. Given the insufficient rehabilitation workforce, telerehabilitation could be encouraged. HBR for children with CP also needs to be popularised in healthcare institutions, including hospitals, rehabilitation centres, and training schools, as well as in communities. Increased awareness is essential for improving the understanding and implementation of HBR and achieving better rehabilitation outcomes in children with CP. CGs should consider seeking more information about CP from health professionals rather than relying on information provided by non-professionals in the community. This will help prevent exposure to misleading information that could hinder progress in rehabilitative care. Finally, physiotherapists and other rehabilitation professionals should consider allocating more time to educating the parents and CGs of children with CP about the different exercises they can practise at home, especially on non-therapy days. Therapists should consider an environment-specific approach when demonstrating these exercises to CGs, as affected children and CGs live in diverse environments requiring different adaptations.

## Conclusions

CGs viewed HBR as convenient, accessible, and empowering. However, they faced substantial challenges, including irregular RT visits, family rejection, and limited skills. RTs encountered difficulties related to the increasing age of the children, insufficient transportation to patients’ homes, and limited institutional permission, which made HBR difficult. As strategies, CGs emphasised the need for additional training, regular home visits by RTs, and home exercise equipment. RTs also highlighted the importance of raising awareness about CP, in addition to the suggestions made by CGs. The findings of this study highlight the need for appropriate health policies on CBR and institutional support for HBR for children with CP. Further studies could adopt a multicentre approach involving several countries to improve understanding of HBR and its determinants across different communities. Factors associated with poor parental or CG adherence to HBR should also be investigated.

## Appendices

Appendix 1

Guided Questionnaires for Caregivers

1. Can you describe your experience with home-based rehabilitation for your child?

2. Which do you prefer: home-based rehabilitation or hospital-based rehabilitation? Please give a reason for your answer.

3. What specific rehabilitation activities do you perform at home?

4. How often do you perform these rehabilitation activities?

5. What resources (e.g., materials or equipment) do you use for home-based rehabilitation?

6. What challenges do you face when implementing home-based rehabilitation?

7. How do these challenges affect your child’s progress?

8. What support do you feel you need to improve your home-based rehabilitation efforts?

9. In what ways have you noticed improvements in your child since starting home-based rehabilitation?

10. How do you perceive the effectiveness of home-based rehabilitation compared with rehabilitation in clinical settings?

11. What advice would you give other caregivers about home-based rehabilitation?

Annexe 1 (français)

Guide d'entretien pour les aidants

1. Pouvez-vous décrire votre expérience de la réadaptation à domicile pour votre enfant ?

2. Laquelle préférez-vous : la réadaptation à domicile ou la réadaptation en milieu hospitalier ? Veuillez justifier votre réponse.

3. Quelles activités spécifiques de réadaptation réalisez-vous à domicile ?

4. À quelle fréquence réalisez-vous ces activités de réadaptation ?

5. Quelles ressources (p. ex., du matériel ou des équipements) utilisez-vous pour la réadaptation à domicile ?

6. Quelles difficultés rencontrez-vous dans la mise en œuvre de la réadaptation à domicile ?

7. Comment ces difficultés affectent-elles les progrès de votre enfant ?

8. De quel soutien pensez-vous avoir besoin pour améliorer vos efforts de réadaptation à domicile ?

9. De quelles manières avez-vous constaté des améliorations chez votre enfant depuis le début de la réadaptation à domicile ?

10. Comment percevez-vous l’efficacité de la réadaptation à domicile par rapport à la réadaptation en milieu clinique ?

11. Quels conseils donneriez-vous à d’autres aidants au sujet de la réadaptation à domicile ?

Appendix 2

Interview Guide for RTs

1. What is your role, and can you describe your experience with home-based rehabilitation for children with CP?

2. Which do you prefer: home-based rehabilitation or hospital-based rehabilitation? Please give a reason for your answer.

3. What specific rehabilitation activities do you perform at home?

4. How often do you perform these rehabilitation activities?

5. What resources (e.g., materials or equipment) do you use for home-based rehabilitation?

6. What challenges do you face when implementing home-based rehabilitation?

7. How do these challenges affect rehabilitation progress?

8. What support do you feel you need to improve your home-based rehabilitation efforts?

9. In what ways have you noticed improvements since starting home-based rehabilitation?

10. How do you perceive the effectiveness of home-based rehabilitation compared with rehabilitation in clinical settings?

Annexe 2 (français)

Guide d'entretien pour les techniciens de réadaptation

1. Quel est votre rôle, et pouvez-vous décrire votre expérience de la réadaptation à domicile pour les enfants atteints de paralysie cérébrale (PC) ?

2. Laquelle préférez-vous : la réadaptation à domicile ou la réadaptation en milieu hospitalier ? Veuillez justifier votre réponse.

3. Quelles activités spécifiques de réadaptation réalisez-vous à domicile ?

4. À quelle fréquence réalisez-vous ces activités de réadaptation ?

5. Quelles ressources (p. ex., du matériel ou des équipements) utilisez-vous pour la réadaptation à domicile ?

6. Quelles difficultés rencontrez-vous dans la mise en œuvre de la réadaptation à domicile ?

7. Comment ces difficultés affectent-elles les progrès de la réadaptation ?

8. De quel soutien pensez-vous avoir besoin pour améliorer vos efforts de réadaptation à domicile ?

9. De quelles manières avez-vous constaté des améliorations depuis le début de la réadaptation à domicile ?

10. Comment percevez-vous l’efficacité de la réadaptation à domicile par rapport à la réadaptation en milieu clinique ?
